# The 22^nd^ annual meeting of the European Tissue Repair Society (ETRS) in Athens, Greece

**DOI:** 10.1186/1755-1536-6-3

**Published:** 2013-02-01

**Authors:** Boris Hinz, Magda Ulrich, Hilde Beele, Dimitris Kletsas

**Affiliations:** 1 Laboratory of Tissue Repair and Regeneration, Matrix Dynamics Group, Faculty of Dentistry, University of Toronto, 150 College Street, Toronto, ON M5S3E2, Canada; 2 Association of Dutch Burn Centres, Beverwijk, The Netherlands and Department of Plastic Reconstructive and Hand Surgery, VU University Medical Center, Amsterdam, The Netherlands; 3 Department of Dermatology and Tissue Bank, Ghent University Hospital, Ghent, Belgium; 4 Laboratory of Cell Proliferation and Ageing, Institute of Biology, National Centre of Scientific Research “Demokritos”, 15310, Athens, Greece

**Keywords:** Tissue repair, Wound healing, Fibrosis, Chronic wounds, Stem cells

## Abstract

The 22^nd^ Annual Meeting of the European Tissue Repair Society, Athens, Greece, October 4 to 5, 2012 informed about pathophysiological mechanisms in tissue repair and on the development of clinical treatments of chronic wounds, fibrosis, and cancer, considering recent advances in molecular biology and biotechnology.

## Introduction

**Figure 1:**
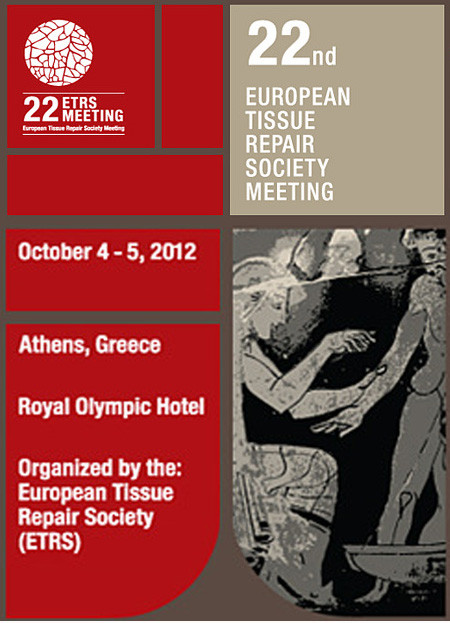
**Official poster announcement of the 22**^**nd **^**Annual Meeting of the European Tissue Repair Society, Athens, Greece, October 4 to 5, 2012.**

Pathological healing is a leading cause of morbidity and mortality despite significant advance of wound care in the 20^th^ century. Severely injured organs cannot always be regenerated by the routine repair mechanisms of the body, thus necessitating the development of novel therapies. The 22^nd^ Annual Meeting of the European Tissue Repair Society (ETRS) was organized by Dimitris Kletsas and colleagues in Athens, Greece, from October 4 to 5, 2012 with almost 200 clinical and fundamental scientist attendees (Figure 1).

The 15 main sessions of the ETRS Annual Meeting [http://www.etrs.org] and the 60 posters on display covered the role of stem cells and cell therapies, growth factors (GF), angiogenesis, mechanical factors, biomaterials and nanotechnology, cell senescence, inflammation, and cell-extracellular matrix (ECM) interactions in tissue repair and regeneration (abstracts published in Wound Repair and Regeneration 2012, Volume 20(5)). Special sessions discussed infections, burns and chronic wounds, systems biology approaches to understand tissue repair, and the similarity between wound repair and cancer development. The ETRS meeting also hosted workshops given by the industry partners Integra and Bruker, and a guest session from the European Wound Management Association (EWMA). The proteomics workshop by Bruker was organized in collaboration with the ETRS Junior Committee, expressing the aim of ETRS to involve and support young scientists. Traditionally, ETRS meetings provide a platform for young investigators and more than 30 talks were selected from early stage researchers, of which nine competed in the Young Investigator’s Award session. This year’s winner was Eirini Apostolou (Biomedical Research Foundation Academy of Athens, Greece), who will be invited to present at the 2013 Annual Meeting of the ETRS partner organization, the Wound Healing Society (WHS), in Denver, Colorado. The next annual meeting of the ETRS will be organized from October 23–25 in Reims, in the beautiful Champagne region of France. Hands-on research on antioxidants is to be expected.

## The clinical impacts of poor and excessive healing

### The Ying of wound healing - chronic wounds

Insufficient healing causes a high level of mortality and morbidity and is an important cost factor for the global health care system. Chronic wounds are present in 2% of the patients in Western countries and 1% worldwide. Chronic wounds particular affect diabetics, physically disabled, elderly and patients with vascular diseases, as was shown in a cross-sectional study performed in India. Finding effective treatments to restore normal healing in chronic wounds and measuring the outcomes are major challenges for clinical therapies, which was discussed in several talks. Risk factors include smoking, whereas smoking cessation and nicotine replacement therapy was shown to improve healing. Other treatments include negative pressure wound therapy (NPWT), and platelet-rich fibrin for general regeneration support.

One major complication in chronic wounds is bacterial infection. Mouse models demonstrate a systemic negative effect of local sepsis on the inflammatory response. Different concepts were presented to target/prevent bacterial infection and biofilm formation, including treatment with local polyhexamethylene biguanidine antiseptics. It was postulated that in some cases, adequately used local antiseptics could prevent the need for systemic antibiotics. Novel concepts involve the use of dextrin-colistin polymers to interfere with biofilm growth and stability and to deliver GFs to the wound bed. Other innovative systems that may improve chronic wound healing include a non-viral piggyback transposon system for gene delivery and nanostructured polymer containers constructed of magnetic nanoparticles to deliver GFs. Such GFs may include Epidermal GF (EGF) to support re-epithelialization or tumor necrosis factor α to control ECM reorganization by matrix metalloproteinases.

Chronic wound healing is often associated with ischemia and with low levels of tissue O_2_ (hypoxia); hypoxia is accepted to be one of the main risk factors for chronic wounds. One session focused on the molecular mechanisms of angiogenesis as targets to support tissue oxygenation. Several biomolecules were addressed, including anti-angiogenic molecules such as advanced glycation end products and pro-angiogenic molecules such as vascular endothelial GF (VEGF), nucleolin, talin and epoxyeicosatrienoic acids. The role of Interleukin-10 was discussed in a talk given by Swati Balaji (Cincinnati Children’s Hospital Medical Center, Cincinnati, OH), the Young Investigator Award winner of the Wound Healing Society’s 2012 Annual Meeting.

### The Yang of wound healing - fibrosis and contractures

Excessive healing involves the excessive and persistent accumulation of ECM, inflammatory cells and reparative cells, which are common in organ fibrosis and skin hypertrophic scarring, such as after large area burns. According to the American Burn Association, approximately 45% of patients with burn wounds died in the period 2001 to 2010 when more than 50% of the skin surface was destroyed. Hypertrophic scars arise from imperfect dermis regeneration and can immobilize digits and limbs; furthermore, the poor esthetics of the scar enormously impact on the patient’s quality of life. A workshop organized by Integra presented ECM restoration strategies to support the formation of a neo-dermis in burns and in post-traumatic and post-surgical wounds.

A number of talks discussed the molecular mechanisms of fibrosis development in other organs and conditions. Deciphering common traits in the pathophysiological processes will be beneficial to develop novel anti-fibrosis strategies. Such approaches include systems biology (for example, gene array meta-analysis of human keloid scars and control tissue, data analysis from a longitudinal human platform to model processes and pathways in variety of pathologies including wound healing). A workshop organized by Bruker informed researchers of the use, principles and instrumentation of proteomic analysis for wound healing research. To identify processes involved in airway scarring, *ex vivo* organ cultures of early non-scarring and late developmental stage scarring fetal rat airways were presented as another model for detecting patterns of pro-fibrotic gene clusters. In-depth analysis of selected pathways and molecules was presented for the pro-fibrotic/wound healing supporting roles of TAK1, a downstream effector of non-Smad transforming GF β1 (TGFβ1) signaling, the β2 adrenoceptor, Trefoil family factor 2 (TFF2) and CCN2 (Connective tissue growth factor, CTGF). The pro-fibrotic cytokine TGFβ was shown to play a major role in normal adult lung repair, whereas Bone morphogenetic protein (BMP) is prevalent during development. The data suggested that during lung injury, BMP is expressed by cells with stem cell characteristics whereas TGFβ hallmarks differentiated cells.

### Inflammation plays a role in chronic wound healing and fibrosis

Inflammatory cells can be both beneficial and detrimental to the outcome of tissue and organ repair. The inflammatory response and the hepatic regeneration potential were compared in animal models of open and laparoscopic left partial hepatectomy. The open surgical procedure resulted in a higher systemic inflammatory response and improved liver regeneration. Viral overexpression of Activin A in mouse lungs leads to chronic inflammation and generates a phenotype that resembles human acute respiratory stress syndrome (ARDS). In another study, histatins were presented to supporting re-epithelialization and wound healing, possibly by modulating the inflammatory response. In chronic wounds, macrophage polarization was presented as a critical event in wound healing. Whereas the pro-inflammatory macrophage M1 type prevails in chronic wounds, a switch to the M2 macrophage appears to improve healing. This switch can be induced by delivered mesenchymal progenitor cells (MSCs).

## The use of multipotent mesenchymal progenitor cells (MSCs) for improved tissue repair

A main focus of the meeting was the use of MSCs to improve tissue repair and regeneration when the body’s repair mechanisms fail. MSCs contribute directly to tissue repair by exerting remodeling activity and by additionally exerting immunosuppressive, trophic, antiseptic, chemotactic, and differentiating effects on other cell populations in the wound environment. Furthermore, MSCs have been proposed as drug and gene-delivery vehicles and a number of methods have been presented to prepare MSCs for implantation purposes into different organs. In addition to the best characterized bone-marrow-derived MSCs, MSCs derived from adipose tissue or oral gingiva were introduced as attractive new cell sources to repair scarring burn skin wounds and chronic wounds, as well as to support the regeneration of diseased liver. For tissue regeneration of the soft palate of the cleft lip, MSCs derived from limb and head muscles were compared with respect to their differentiation potential. Moreover, bulge cells derived from hair follicles have been shown to provide a potential source of cells for wound repair. In addition to presenting the scientific and clinical potential of MSCs, practical aspects were discussed at the meeting, such as tissue and cell banking and drawbacks of the changing EU legislation concerning the use of stem cells.

## The microenvironment affects the regenerative potential of reparative cells

The meeting sensitized all attendees to the fact that the chemical and mechanical conditions present in different injured tissues are important determinants of the cellular repair process and have to be considered for the development of treatment strategies for tissue regeneration. In cartilage, steep gradients of oxygen, glucose, and pH between the surface and the interior are generated due to the avascular nature of the tissue. Different dental scaffolds for tooth repair were shown to have different suitability for use with different oral-derived MSCs. In skin regeneration, it is important to suppress potential fibrotic characteristics of MSCs induced by expansion cell culture. Similarly, culture-induced senescence of MSCs was flagged as being detrimental for wound healing and tissue regeneration because of the pro-inflammatory state of senescent MSCs.

The mechanical conditions present in tissue under repair, such as strain and ECM stiffness, were highlighted as particularly important factors for the repair success. Extracellular proteins including Periostin and Cartilage oligomeric matrix protein (COMP) provide cell information on the physical state of the ECM. This mechanical information is perceived through integrin containing cell-ECM adhesions. The specific roles of different integrins were discussed for epithelial and fibroblast cell differentiation and different knock-out models were phenotyped. Cell-ECM adhesions and transmembrane integrins are intracellularly linked to the actin/myosin cytoskeleton. It was shown that cell shape, dictated by the ECM configuration, regulates internal stress and the polymerization degree of actin. In turn, the content of globular actin controls the translocation of the myocardin-related transcription factor MRTF from the cytosol to the nucleus and thus, stress-dependent gene expression. Another mechanotransduction pathway involves the activation of the JAK/STAT pathway, and a new link between the mechanical activation of TGFβ1 and ECM stiffness was revealed.

Modulation of mechanical stress is clinically exploited to improve chronic wound healing. With more than 20 different NPWT systems on the market, the search for their mechanisms of action has revealed mechanical stress as a critical component of NPWT therapy success. Negative pressure induces tissue macro- and micro-deformations and fluid shear forces that impact the activity of fibroblasts, blood vessels and lymphatics, in combination with the material properties of the NPWT foam.

## The similarities between tissue repair and cancer

Cancer development is similarly driven by the mechanical properties present in the stroma. The similarities in growth factor signaling, neo-vascularization and tissue remodeling between cancer and wound healing was addressed in the Charles Lapière memorial keynote lecture given by Dr. Sabine Werner (Eidgenössische Technische Hochschule Zürich, Switzerland). Dr. Werner focused on three recently studied proteins in cancer and wound healing: 1) perioxyredoxin 6 seems to protect skin from development of benign tumors by controlling lipid and protein oxidation but conversely promotes transition of benign to malignant tumors by protecting cancer cells from oxidative stress; 2) the transcription factor Nrf3 is downregulated during epidermal healing in keratinocytes and seems to be a negative regulator of normal healing; and 3) the transcription factor Nrf2 is highly expressed in suprabasal keratinocytes in the skin epidermis and regulates the antioxidant defense system. Constitutively active Nrf2 expressed under a keratinocyte-specific promoter protects from tumorigenesis but leads to ichtyosis and hyperkeratosis. The potential of pharmacological activators of Nrf2 in affecting tumorigenesis is currently under investigation. A number of other talks underlined that various ECM molecules which are important during wound healing influence cancer malignancy; in turn, cancer cells modulate the expression of ECM molecules in the stroma. For instance, paracrine factors released by breast cancers cells stimulate endothelial cells to express hyaluronic acid, its receptor CD44 and the adhesion molecules VCAM and ICAM. In turn, MT1-matrix metalloproteinase expression and endothelial cell migration are reduced. Collectively, it became evident at the meeting that knowledge and therapies generated in the field of cancer research are of tremendous use for chronic and excessive wound healing and vice versa.

## Abbreviations

ARDS: Acute respiratory stress syndrome; BMP: Bone morphogenetic protein; COMP: Cartilage oligomeric matrix protein; ECM: Extracellular matrix; EGF: Epidermal growth factor; ETRS: European Tissue Repair Society; EWMA: European Wound Management Association; GF: Growth factor; MRTF: Myocardin-related transcription factor; MSC: Mesenchymal stromal cell; NPWT: Negative pressure wound therapy; TGF: Transforming growth factor; TFF2: Trefoil family factor 2; VEGF: Vascular endothelial growth factor; WHS: Wound Healing Society.

## Competing interests

The authors state that they have no competing interests.

## Authors’ contributions

BH contributed to the writing of 50% and coordinated the manuscript. MU, HB and DK contributed to the other 50%. All authors have read and approved the final manuscript.

